# Supplementary material for: Circulatory MicroRNAs in Plasma and Atrial Fibrillation in the General Population: The Rotterdam Study
Source: Genes (Basel). 2021 Dec 22;13(1):11. doi: 10.3390/genes13010011 (PMC8775308; doi:10.3390/genes13010011)
Supplement: Supplementary file 1 [file genes-13-00011-s001.zip › genes-1483954-supplementary.pdf]

SUPPLEMENTARY MATERIAL

Methods S1. Study population

Methods S2. Assessment of circulatory microRNAs in plasma

Methods S3. Assessment of atrial fibrillation

Methods S4. Assessment of cardiovascular risk factors

Table S1. Baseline characteristics of the total study population and stratified by sex

Table S2. MicroRNAs nominally associated with the odds of prevalent atrial fibrillation in the total study population and stratified by sex

Table S3. MicroRNAs nominally associated with the risk of incident atrial fibrillation in the total study population and stratified by sex

Table S4. Predicted target genes of miR-4798-3p that have been previously associated with atrial fibrillation and their potential remodeling mechanism

Table S5. Circulatory microRNAs reported in previous literature in association with atrial fibrillation

Table S6. Association of previously-reported microRNAs with the odds of prevalent atrial fibrillation in our total study population and stratified by sex

Table S7. Association of previously-reported microRNAs with the risk of incident atrial fibrillation in our total study population and stratified by sex

Methods S1. Study population

The Rotterdam Study is a prospective population-based cohort study that investigates the occurrence and progression of risk factors for chronic diseases in middle-age and elderly persons [26,27]. The Rotterdam study started in 1990 and all inhabitants aged ≥55 years of Ommoord district in the city of Rotterdam in The Netherlands were invited. A total of 7983 (78% of all invitees) inhabitants participated (RS-I). The cohort was extended in 2000 with 3011 participants who were ≥55 years or who had migrated to Ommoord (RS-II). The cohort was again extended in 2006 with 3932 participants that were ≥45 years (RS-III). The overall response rate was 72% at baseline. All participants were re-examined every 3-5 years at the research center. Data on morbidity and mortality were continuously collected through linkage with medical files from general practitioners in the study area [26,27].

For the present study, we included 1000 participants from the fourth visit of RS-I (RS-I-4) and 1000 participants from the second visit of RS-II (RS-II-2) for whom miRNA expression data was obtained (n=2000). These visits took place between 2002 and 2005 which we considered as the baseline of our study. From these 2000 randomly chosen participants, one participant was excluded, because of insufficient baseline data on AF for the cross-sectional study. For the longitudinal study with incident AF, we additionally excluded prevalent AF cases (n=98). A total of 1999 participants were included in the current study.

The Rotterdam Study complies with the Declaration of Helsinki and has been approved by the Medical Ethics Committee of the Erasmus Medical Center (registration number MEC 02.1015) and by the Dutch Ministry of Health, Welfare and Sport (Population Screening Act WBO, license number 1071272-159521-PG). The Rotterdam Study Personal Registration Data collection is filed with the Erasmus MC Data Protection Officer under registration number

EMC1712001. The Rotterdam Study has been entered into the Netherlands National Trial Register (NTR; www.trialregister.nl/trials) and into the WHO International Clinical Trials Registry Platform (ICTRP; https://apps.who.int/trialsearch/) under shared catalogue number NL6645/NTR6831. All participants provided written informed consent to participate, prior to inclusion, in the study and to have their information obtained from treating physicians.

Methods S2. Assessment of circulatory microRNAs in plasma

Plasma miRNA levels were obtained between 2002 and 2005 in 1000 randomly selected participants from the fourth visit of RS-I (RS-I-4) and 1000 randomly selected participants from the second visit of RS-II (RS-II-2). Plasma miRNA levels were determined using the HTG EdgeSeq miRNA Whole Transcriptome Assay (WTA), which measures the expression levels of 2083 mature human miRNAs (HTG Molecular Diagnostics, Tuscon, AZ, USA) and using the Illumina NextSeq 500 sequencer (Illumina, San Diego, CA, USA). The quantification of miRNA expression was based on counts per million. As standardization and adjustment for total reads within each sample a Log2 transformation of counts per million was used. MiRNAs with a Log2 counts per million <1.0 were indicated as not expressed. A lower limit of quantification was used to select well-expressed miRNAs. Well-expressed miRNAs in plasma were those miRNAs with >50% values above the lower limit of quantification. Out of 2083 measured miRNAs, 591 miRNAs were well-expressed in plasma [28].

Methods S3. Assessment of atrial fibrillation

AF was defined in accordance with the European Society of Cardiology (ESC) guidelines [7].

A 10-second 12-lead electrocardiogram (ECG) was used to assess AF at baseline and follow-up examinations with an ACTA Gnosis IV ECG recorder (Esaote; Biomedica, Florence Italy). The ECG records were stored digitally, and analyzed with the Modular ECG Analysis system (MEANS) [29]. The ECG records diagnosed by MEANS as rhythm disorder were independently verified by two research physicians blinded to the MEANS diagnosis [3,26]. A cardiologist was consulted in case of disagreement between the research physicians. Events of AF were not included if these occurred during the process of dying, or in case of transient AF after cardiac surgery or myocardial infarction [3,26]. Additional follow-up information was obtained from medical files of participating general practitioners, outpatient clinics, hospitals, a national registration of all hospitals discharge diagnoses and follow-up examinations at the research center. The date of incident AF was defined as the date of the first occurrence of symptoms suggestive of AF with subsequent electrocardiogram (ECG) verification obtained from the medical records. All participants were followed from the date of enrollment in the Rotterdam Study until the date of onset of AF, date of death, loss to follow-up, or to January 1st, 2014, whichever occurred first.

Methods S4. Assessment of cardiovascular risk factors

Information on current health status, medical history, medication, and life style was obtained using computerized questionnaires. Participants were interviewed at home by trained interviewers, and underwent clinical examination and laboratory blood sampling at the research center [26,27].

Body mass index (BMI) was calculated as weight (in kg) divided by height (in cm) squared. Serum total and high-density lipoprotein (HDL) cholesterol were measured with an

automated enzymatic method. Blood pressure was measured twice at the right upper arm with a random zero mercury sphygmomanometer in the sitting position. Systolic and diastolic blood pressures were calculated as the mean of the two consecutive measurements. Hypertension was defined as a systolic blood pressure of ≥140 mmHg or a diastolic blood pressure ≥90 mmHg or use of antihypertensive drugs prescribed for hypertension [3]. Smoking information derived from baseline questionnaires was categorized into never, former, and current smokers. Diabetes mellitus (DM) was defined as fasting serum glucose levels ≥7.0 mmol/L (126 mg/dL) (or non-fasting serum glucose levels ≥11.1 mmol/L (200 mg/dL) if fasting samples were unavailable) or the use of antidiabetic therapy. The assessment and definition of history of coronary heart disease (CHD), and heart failure (HF) has been described in detail previously [26]. Left ventricular hypertrophy (LVH) on electrocardiogram (ECG) was diagnosed using the MEANS program with an algorithm that takes into account QRS voltages, with an age-dependent correction and repolarization. Medication use was derived from baseline questionnaires, pharmacy data and was categorized and defined according to the World Health Organization Anatomical Therapeutic Chemical (WHO ATC) classifications. More specifically, cardiac medication, antihypertensive medication, and lipid lowering medication were defined according to the WHO ATC categories c01, c02, and c10 respectively.

Table S1. Baseline characteristics of the total study population and stratified by sex

Baseline Characteristics *	Total Study Population<br>n=1901	Men<br>n=800	Women<br>n=1101	*p*-value †
Age, years	71.4 ± 7.5	71.0 ± 7.1	71.7 ± 7.7	0.022
Women, n (%)	1101 (57.9)	NA	1101 (100)	
Body mass index, kg/m^2	27.7 ± 4.1	27.6 ± 3.4	27.7 ± 4.6	0.582
Total serum cholesterol, mmol/L ‡	5.7 ± 1.0	5.4 ± 1.0	5.9 ± 1.0	<0.001
High-density lipoprotein cholesterol, mmol/L ‡	1.4 ± 0.4	1.3 ± 0.3	1.6 ± 0.4	<0.001
Hypertension, n (%)	1469 (77.3)	604 (75.5)	865 (78.6)	0.115
Smoking status, n (%)				<0.001
Never	572 (30.1)	112 (14.0)	460 (41.7)	
Former	1031 (54.2)	545 (68.1)	486 (44.1)	
Current	298 (15.7)	143 (17.9)	155 (14.1)	
History of diabetes mellitus, n (%)	248 (13.0)	129 (16.1)	119 (10.8)	0.001
History of coronary heart disease, n (%)	188 (9.9)	121 (15.1)	67 (6.1)	<0.001
History of heart failure, n (%)	83 (4.4)	37 (4.6)	46 (4.2)	0.638
Left ventricular hypertrophy, n (%)	95 (5.0)	50 (6.3)	45 (4.1)	0.033
Cardiac medication, n (%)	168 (8.8)	76 (9.5)	92 (8.4)	0.100
Lipid lowering medication, n (%)	419 (22.0)	191 (23.9)	228 (20.7)	0.386

Abbreviations: n, number; NA, not applicable.

The table presents baseline characteristics of the total study population for the analyses of incident atrial fibrillation

* Values are mean (standard deviation) or number (percentages).

† Statistical significance between men and women for continuous data was tested using the Student's T-test and for categorical data was tested using the Chi Square Test.

‡ SI conversion factors: To convert cholesterol to mg/dL divide by 0.0259.

Table S2. MicroRNAs nominally associated with the odds of prevalent atrial fibrillation in the total study population and stratified by sex

Total Study Population			Men			Women		
**miRNA**	**OR (95% CI) ***	***p*-value**	**miRNA**	**OR (95% CI) ***	***p*-value**	**miRNA**	**OR (95% CI) ***	***p*-value**
miR-122-5p	1.42 (1.12-1.78)	0.002967	miR-4798-3p	0.39 (0.24-0.66)	0.000248	miR-1273h-5p	2.55 (1.36-4.89)	0.004270
miR-8078	1.54 (1.16-2.06)	0.003384	miR-122-5p	1.77 (1.28-2.46)	0.000539	miR-6788-3p	0.55 (0.37-0.86)	0.004616
miR-194-5p	1.82 (1.21-2.74)	0.004300	miR-4798-5p	0.47 (0.30-0.77)	0.001531	miR-1273d	3.06 (1.45-6.82)	0.004675
miR-1322	2.01 (1.24-3.32)	0.005358	miR-605-3p	0.50 (0.32-0.80)	0.002291	miR-1304-3p	5.56 (1.74-18.97)	0.004847
miR-378e	0.49 (0.30-0.83)	0.006478	miR-4784	0.48 (0.30-0.82)	0.003185	miR-548ay-5p	4.81 (1.65-15.27)	0.005846
miR-1254	1.77 (1.17-2.69)	0.007442	miR-192-5p	2.38 (1.35-4.29)	0.003233	miR-8078	1.97 (1.21-3.32)	0.008302
miR-574-3p	1.99 (1.19-3.31)	0.008025	miR-4721	0.57 (0.39-0.86)	0.003958	miR-1322	3.04 (1.36-7.10)	0.008354
miR-4721	0.66 (0.50-0.92)	0.008546	miR-194-5p	2.29 (1.29-4.11)	0.005029	miR-1231	0.53 (0.33-0.87)	0.009599
miR-6852-5p	0.54 (0.34-0.88)	0.009831	miR-4512	2.74 (1.37-5.70)	0.005538	miR-574-3p	2.94 (1.24-6.98)	0.014333
miR-4798-5p	0.60 (0.42-0.91)	0.010017	miR-6747-3p	2.41 (1.30-4.68)	0.007422	miR-5585-3p	2.36 (1.22-4.84)	0.014432
miR-1909-3p	1.51 (1.10-2.07)	0.010265	miR-6794-5p	0.15 (0.03-0.67)	0.012026	miR-6797-5p	0.64 (0.45-0.95)	0.017631
miR-204-3p	0.36 (0.16-0.80)	0.011161	miR-2116-5p	0.42 (0.22-0.89)	0.012516	miR-6500-3p	1.89 (1.15-3.37)	0.020219
miR-1285-5p	1.58 (1.11-2.27)	0.012415	miR-125a-5p	0.41 (0.21-0.87)	0.015047	miR-1285-5p	2.06 (1.14-3.89)	0.020352
miR-1273d	1.71 (1.13-2.65)	0.013537	miR-3124-3p	0.43 (0.22-0.88)	0.016097	miR-1254	2.30 (1.13-4.71)	0.021772
miR-4512	1.84 (1.16-3.03)	0.013550	miR-3667-5p	0.48 (0.28-0.92)	0.016510	miR-1273e	2.06 (1.13-3.88)	0.022274
miR-5585-3p	1.63 (1.11-2.43)	0.014015	miR-4723-3p	0.39 (0.18-0.89)	0.017808	miR-378e	0.43 (0.21-0.94)	0.024289
miR-192-5p	1.64 (1.11-2.45)	0.014723	miR-335-5p	0.54 (0.32-0.91)	0.020447	miR-566	2.73 (1.19-6.91)	0.025762
miR-1304-3p	2.22 (1.17-4.30)	0.016146	miR-10b-5p	0.45 (0.23-0.93)	0.021481	miR-4421	2.09 (1.14-4.16)	0.026404
miR-1255b2-3p	1.54 (1.08-2.18)	0.017186	miR-3912-5p	0.36 (0.16-0.91)	0.022020	miR-1255b2-3p	1.95 (1.08-3.51)	0.026521
miR-4784	0.61 (0.41-0.95)	0.019058	miR-4734	2.16 (1.08-4.07)	0.022058	miR-1273a	2.23 (1.13-4.68)	0.026830
miR-616-3p	1.62 (1.10-2.48)	0.019789	miR-6715b-3p	0.42 (0.20-0.91)	0.023262	miR-213p	0.47 (0.25-0.94)	0.027541
miR-4421	1.54 (1.08-2.23)	0.019957	miR-30a-5p	0.34 (0.14-0.89)	0.023364	miR-1273c	2.49 (1.10-5.65)	0.028319
miR-1273h-5p	1.54 (1.07-2.25)	0.020961	miR-3687	1.98 (1.05-3.54)	0.027593	miR-1269b	2.10 (1.13-4.25)	0.028337
miR-29c-5p	0.69 (0.51-0.96)	0.021375	miR-4713-3p	0.63 (0.42-0.98)	0.030730	miR-548d-5p	2.18 (1.08-4.50)	0.031964
miR-3687	1.67 (1.06-2.58)	0.022307	miR-1909-3p	1.58 (1.05-2.41)	0.030829	miR-3674	2.31 (1.09-5.07)	0.032957
miR-141-3p	0.51 (0.29-0.93)	0.022368	miR-100-5p	1.99 (1.09-4.00)	0.031450	miR-3135a	2.26 (1.06-4.83)	0.035470

miR-6794-5p	0.28 (0.09-0.85)	0.023037	miR-670-3p	0.38 (0.16-0.95)	0.032241	miR-4539	2.03 (1.05-3.98)	0.037016
miR-6788-3p	0.70 (0.52-0.97)	0.023570	miR-920	0.46 (0.23-0.96)	0.032879	miR-616-3p	2.19 (1.10-4.89)	0.039789
miR-3674	1.63 (1.07-2.51)	0.024082	miR-4695-5p	1.70 (1.01-2.74)	0.034056	miR-765	0.57 (0.34-1.02)	0.041683
miR-566	1.74 (1.08-2.87)	0.026028	miR-133a-5p	0.45 (0.22-0.99)	0.036036	miR-1539	2.02 (1.12-4.30)	0.041994
miR-765	0.64 (0.43-0.96)	0.027041	miR-6761-5p	0.52 (0.29-0.99)	0.037263	miR-4459	2.55 (0.98-6.29)	0.048123
miR-4753-5p	0.49 (0.27-0.95)	0.027462	miR-4543p	0.59 (0.36-0.99)	0.039182			
miR-5684	1.41 (1.05-1.97)	0.029441	miR-4319	0.55 (0.32-1.01)	0.039455			
miR-1273e	1.49 (1.04-2.15)	0.030004	miR-1365p	0.81 (0.66-1.00)	0.040095			
miR-7106-5p	0.71 (0.52-0.98)	0.032349	miR-193a-5p	0.49 (0.25-0.96)	0.040877			
miR-4798-3p	0.63 (0.42-0.99)	0.034433	miR-362-5p	0.64 (0.43-1.00)	0.041871			
miR-6877-3p	2.12 (1.06-4.31)	0.034941	miR-6791-5p	1.48 (1.01-2.15)	0.043547			
miR-6716-3p	0.57 (0.34-0.99)	0.035978	miR-155-5p	0.59 (0.36-1.00)	0.043632			
miR-4426	0.76 (0.59-0.99)	0.036714	miR-548ay-5p	0.58 (0.36-1.04)	0.045788			
miR-3667-5p	0.63 (0.42-1.01)	0.038123	miR-2116-3p	0.39 (0.16-1.05)	0.045877			
miR-1228-3p	1.93 (1.04-3.66)	0.041108	miR-196b-3p	0.51 (0.26-1.00)	0.047484			
miR-1303	1.58 (1.02-2.47)	0.041760	miR-4722-3p	1.86 (1.00-3.39)	0.047545			
miR-1273c	1.62 (1.01-2.57)	0.042953	miR-217	0.49 (0.25-1.03)	0.048351			
miR-6887-5p	0.71 (0.51-0.99)	0.043152	let-7d-3p	0.49 (0.24-1.00)	0.048457			
miR-1269b	1.44 (1.03-2.08)	0.043577	miR-6887-5p	0.64 (0.41-1.00)	0.049277			
miR-6747-3p	1.53 (1.02-2.36)	0.047232						
miR-30a-5p	0.48 (0.23-1.02)	0.049055						

Abbreviations: CI, confidence interval; OR, odds ratio.

* Adjusted for age, cohort, body mass index, total cholesterol, high-density lipoprotein cholesterol, hypertension, smoking status, history of diabetes, history of coronary heart disease, history of heart failure, left ventricular hypertrophy on the electrocardiogram, use of cardiac medication, and use of lipid lowering medication (model 2).

Table S3. MicroRNAs nominally associated with the risk of incident atrial fibrillation in the total study population and stratified by sex

**Total Study Population**			**Men**			**Women**		
**miRNA**	**HR (95% CI) ***	***p*-value**	**miRNA**	**HR (95% CI) ***	***p*-value**	**miRNA**	**HR (95% CI) ***	***p*-value**
miR-197-3p	0.58 (0.40-0.84)	0.003531	miR-378e	0.43 (0.26-0.71)	0.001137	miR-197-3p	0.47 (0.29-0.79)	0.003940
miR-548w	1.38 (1.10-1.74)	0.006042	miR-6769b-3p	4.22 (1.51-11.74)	0.005900	miR-181b-5p	0.56 (0.37-0.84)	0.005111
miR-6765-5p	1.54 (1.08-2.20)	0.017530	miR-1290	0.67 (0.49-0.90)	0.008000	miR-185-3p	0.78 (0.64-0.94)	0.010826
miR-6894-5p	0.75 (0.59-0.97)	0.025632	miR-7111-3p	0.69 (0.52-0.91)	0.008684	miR-181d-5p	0.81 (0.68-0.96)	0.017475
miR-548b-5p	1.24 (1.02-1.51)	0.033402	miR-5006-5p	2.61 (1.24-5.49)	0.011750	miR-548w	1.53 (1.08-2.18)	0.018272
miR-6126	1.17 (1.01-1.36)	0.033909	miR-4270	0.58 (0.38-0.89)	0.013263	miR-561-5p	1.98 (1.11-3.53)	0.019960
miR-3197	0.89 (0.79-0.99)	0.036662	miR-4758-5p	0.61 (0.40-0.92)	0.019000	miR-6765-5p	1.82 (1.06-3.12)	0.028780
miR-324-3p	0.70 (0.49-0.98)	0.038061	miR-532-5p	0.65 (0.46-0.93)	0.019790	miR-3689a-3p	0.81 (0.67-0.98)	0.031271
miR-1307-5p	1.32 (1.01-1.73)	0.039834	miR-378a-3p	0.53 (0.31-0.91)	0.021570	miR-204-3p	2.50 (1.08-5.77)	0.032405
miR-17-3p	0.69 (0.48-0.99)	0.041655	miR-6500-3p	0.80 (0.66-0.97)	0.023000	miR-125a-5p	0.55 (0.31-0.97)	0.038775
miR-6715b-3p	1.57 (1.02-2.43)	0.041864	miR-7978	1.74 (1.07-2.81)	0.024209	miR-4676-3p	1.54 (1.02-2.33)	0.041641
miR-1287-5p	1.23 (1.01-1.51)	0.042095	miR-4279	2.20 (1.09-4.44)	0.027113	miR-3169	1.41 (1.01-1.96)	0.042371
miR-4800-5p	0.73 (0.54-0.99)	0.044090	miR-6892-3p	1.82 (1.07-3.10)	0.027160	miR-324-3p	0.60 (0.36-0.99)	0.043760
miR-4522	1.32 (1.01-1.72)	0.044800	miR-3157-3p	2.39 (1.08-5.29)	0.031678			
miR-658	1.40 (1.01-1.94)	0.044996	miR-2115-3p	3.15 (1.10-9.02)	0.032931			
miR-4449	0.72 (0.52-0.99)	0.045036	miR-299-5p	1.54 (1.03-2.29)	0.034224			
miR-93-5p	0.71 (0.51-0.99)	0.046484	miR-4646-3p	0.75 (0.57-0.98)	0.035213			
			miR-149-3p	1.42 (1.02-1.98)	0.035383			
			miR-122-5p	0.75 (0.57-0.98)	0.036170			
			miR-2276-3p	1.62 (1.03-2.55)	0.037520			
			miR-4644	1.30 (1.02-1.66)	0.037586			
			miR-658	1.64 (1.03-2.62)	0.038120			
			miR-6742-5p	2.05 (1.04-4.06)	0.039417			
			miR-4309	2.05 (1.01-4.16)	0.046510			
			miR-1287-5p	1.34 (1.00-1.79)	0.046800			

			miR-1229-3p	0.81 (0.66-1.00)	0.047938				

Abbreviations: CI, confidence interval; HR, hazard ratio.

* Adjusted for age, cohort, body mass index, total cholesterol, high-density lipoprotein cholesterol, hypertension, smoking status, history of diabetes, history of coronary heart disease, history of heart failure, left ventricular hypertrophy on the electrocardiogram, use of cardiac medication, and use of lipid lowering medication (model 2).

Table S4. Predicted target genes of microR-4798-3p that have been previously associated with atrial fibrillation and their potential remodeling mechanism

Predicted target gene	Target gene name	Remodeling mechanism
CACNB2	Calcium channel, voltage-dependent, beta 2 subunit [43].	Electrical.
KCNN3	Potassium intermediate/small conductance calcium-activated channel, subfamily N, member 3 [44].	Electrical.
SIRT1	Sirtuin 1 [45].	Structural.
STAT3	Signal transducer and activator of transcription 3 [46,47].	Structural.
SLIT3	Slit homolog 3 [48].	Structural.
NAV2	Neuron navigator 2 [48].	Structural.
MYOCD	Myocardin [48].	Structural.
PHLDA1	Pleckstrin homology-like domain, family A, member 1 [38].	Structural.
REEP1	Receptor accessory protein 1 [38].	Structural.
FAM13B	Family with sequence similarity 13, member B [38].	Structural.
ATXN1	Ataxin 1-like [38].	Structural.
UST	Uronyl-2-sulfotransferase [38].	Structural.
CDK6	Cyclin-dependent kinase 6 [38].	Structural.
CAV2	Caveolin 2 [38].	Structural.
XPO7	Exportin 7 [38].	Structural.
FBXO32	F-box protein 32 [38].	Structural.
FUT11	Fucosyltransferase 11 [38].	Structural.
ASAH1	N-acylsphingosine amidohydrolase 1 [38].	Structural.
ORMDL3	ORM1-like 3 [38].	Structural.
WNT3	Wingless-type MMTV integration site family, member 3 [38].	Structural.
USP36	Ubiquitin specific peptidase 36 [38].	Structural.

Table S5. Circulatory microRNAs reported in previous literature in association with atrial fibrillation

miRNA	Study design	Study population	Characteristics	Reported effect estimates	Model and adjustments
**let-7b-5p**	Prospective patient-based cohort study [21].	Patients enrolled in the miRhythm Study who were evaluated for treatment and that underwent ablation if needed (no AF vs. AF).	n=211 patients (52.6% AF), mean age 58.6 ± 11.9 years, 41.7% women.	Plasma concentrations associated with prevalent AF. OR: 0.48, 95% CI: 0.38-0.61 ($p=3.6x10^{-10}$)<br><br>Bonferroni p=0.0006 (0.05/86 miRNAs).	Multivariable logistic regression. Model: age, sex, current smoking, diabetes, prevalent heart failure, and myocardial infarction.
**let-7b-5p**	Prospective population-based cohort study [24].	Participants who were enrolled in Framingham Heart Study.	n=1788 participants (4.7% prevalent AF and 8.8% incident AF), mean age 66 ± 9 years, 54.4% women. Median follow-up 8.6 years.	Plasma concentrations associated with prevalent AF. OR: 1.27, 95% CI: 1.02-1.59 (p=0.034).	Multivariable logistic regression. Model: age, sex, current smoking, diabetes, prevalent heart failure, and myocardial infarction. Bonferroni p=0.000147 (0.05/340 miRNAs).<br>Model and Bonferroni correction were used in step 1 of the analyses to identify 73 microRNAs and these 73 miRNAs were used for the second analysis.
**let-7c-5p**	Prospective patient-based cohort study [21].	Patients enrolled in the miRhythm Study who were evaluated for treatment and that underwent ablation if needed (no AF vs. AF).	n=211 patients (52.6% AF), mean age 58.6 ± 11.9 years, 41.7% women.	Plasma concentrations associated with prevalent AF. OR: 0.47, 95% CI: 0.37-0.59 ($p=2.5x10^{-10}$)<br><br>Bonferroni p=0.0006 (0.05/86 miRNAs).	Multivariable logistic regression. Model: age, sex, current smoking, diabetes, prevalent heart failure, and myocardial infarction.
**let-7d-5p**	Prospective population-based cohort study [24].	Participants who were enrolled in Framingham Heart Study.	n=1788 participants (4.7% prevalent AF and 8.8% incident AF), mean age 66 ± 9 years, 54.4% women. Median follow-up 8.6 years.	Plasma concentrations associated with prevalent AF. OR: 1.29, 95% CI: 1.04-1.6 (p=0.023).	Multivariable logistic regression. Model: age, sex, current smoking, diabetes, prevalent heart failure, and myocardial infarction. Bonferroni p=0.000147 (0.05/340 miRNAs).<br>Model and Bonferroni correction were used in step 1 of the analyses to identify 73 microRNAs and these 73 miRNAs were used for the second analysis.
**RNU48-a2**	Prospective population-based cohort study [20].	Participants who were enrolled in Framingham Heart Study.	n=2445 participants (6.2% prevalent AF and 6.6% incident AF), mean age 66.3 ± 8.9 years, 56.0% women. Median follow-up 5.4 years.	1.10-fold increased plasma concentrations in AF vs. SR (p=NR).<br>Plasma concentrations associated with prevalent AF.<br>Model 1: OR: 1.29, 95% CI: 1.08-1.53 (p=0.005)<br>Model 2: OR: 1.28, 95% CI: 1.03-1.59 (p=0.027).<br><br>Bonferroni $p=2.0\times10^{-4}$ (0.05/253 miRNAs).	Multivariable logistic regression. Model 1: age, sex, miR-processing.<br>Model 2: age, sex, miR-processing, height, weight, systolic blood pressure, diastolic blood pressure, current smoking, antihypertensive medication use, prevalent heart failure, myocardial infarction, and diabetes mellitus.

**miR-1**	Prospective patient-based cohort study [23].	Patients that underwent on-pump CABG without history of AF were monitored for POAF.	n=42 patients (16.7% POAF), mean age 65.0 ± 1.3 years, 26.2% women. Follow-up to 7 days after surgery by continuous ECG monitoring.	No difference in plasma concentrations between POAF vs. SR (p=NS).	NA.
**miR-1**	Cross-sectional Patient-based case-control study [22].	Patients that underwent ablation for AF were matched with WPW patients as controls.	n=40 patients (75.0% AF), mean age 63.7 ± 2.3 years, 30.0% women.	No difference in plasma concentrations between AF vs. SR (p=NS).	NA.
**miR-10b-5p**	Prospective patient-based cohort study [21].	Patients enrolled in the miRhythm Study who were evaluated for treatment and that underwent ablation if needed (no AF vs. AF).	n=211 patients (52.6% AF), mean age 58.6 ± 11.9 years, 41.7% women.	Plasma concentrations associated with prevalent AF. OR: 0.49, 95% CI: 0.39-0.62 ($p=1.9x10^{-9}$).<br><br>Bonferroni p=0.0006 (0.05/86 miRNAs).	Multivariable logistic regression. Model: age, sex, current smoking, diabetes, prevalent heart failure, and myocardial infarction.
**miR-15**	Cross-sectional Patient-based case-control study [16].	Patients from outpatient clinic (no AF vs. AF no HF vs. no AF with HF and vs. AF, HF).	n=95 patients (34.7% AF), mean age 66.4 ± 1.7 years, 18.9% women.	No difference in plasma concentrations between AF vs. SR (p=NS). Plasma concentrations were not associated with prevalent AF. Beta: 1.9, 95% CI: -1.3 - 5.1 (p=0.23).	Multivariable linear regression. Model: age, sex, coronary artery disease, hypertension, diabetes mellitus, hypercholesterolemia, use of angiotensin converting enzyme inhibitor or AT1-receptor blocker, aldosterone antagonists, and/or statins.
**miR-15b-5p**	Prospective population-based cohort study [24].	Participants who were enrolled in Framingham Heart Study.	n=1788 participants (4.7% prevalent AF and 8.8% incident AF), mean age 66 ± 9 years, 54.4% women. Median follow-up 8.6 years.	Plasma concentrations associated with prevalent AF. OR: 1.31, 95% CI: 1.08-1.59 (p=0.007).	Multivariable logistic regression. Model: age, sex, current smoking, diabetes, prevalent heart failure, and myocardial infarction. Bonferroni p=0.000147 (0.05/340 miRNAs). Model and Bonferroni correction were used in step 1 of the analyses to identify 73 microRNAs and these 73 miRNAs were used for the second analysis.
**miR-17-5p**	Prospective population-based cohort study [24].	Participants who were enrolled in Framingham Heart Study.	n=1788 participants (4.7% prevalent AF and 8.8% incident AF), mean age 66 ± 9 years, 54.4% women. Median follow-up 8.6 years.	Plasma concentrations associated with prevalent AF. OR: 1.24, 95% CI: 1.03-1.5 (p=0.023).	Multivariable logistic regression. Model: age, sex, current smoking, diabetes, prevalent heart failure, and myocardial infarction. Bonferroni p=0.000147 (0.05/340 miRNAs). Model and Bonferroni correction were used in step 1 of the analyses to identify 73 microRNAs and these 73 miRNAs were used for the second analysis.
**miR-19a-3p**	Prospective population-based cohort study [24].	Participants who were enrolled in Framingham Heart Study.	n=1788 participants (4.7% prevalent AF and 8.8% incident AF), mean age 66 ± 9 years, 54.4%	Plasma concentrations associated with prevalent AF. OR: 1.18, 95% CI: 1-1.39 (p=0.048).	Multivariable logistic regression. Model: age, sex, current smoking, diabetes, prevalent heart failure, and myocardial infarction.

			women. Median follow-up 8.6 years.		Bonferroni p=0.000147 (0.05/340 miRNAs).<br>Model and Bonferroni correction were used in step 1 of the analyses to identify 73 microRNAs and these 73 miRNAs were used for the second analysis.
**miR-20a-5p**	Prospective population-based cohort study [24].	Participants who were enrolled in Framingham Heart Study.	n=1788 participants (4.7% prevalent AF and 8.8% incident AF), mean age 66 ± 9 years, 54.4% women. Median follow-up 8.6 years.	Plasma concentrations associated with prevalent AF. OR: 1.36, 95% CI: 1.14-1.61 (p=0.001).	Multivariable logistic regression. Model: age, sex, current smoking, diabetes, prevalent heart failure, and myocardial infarction. Bonferroni p=0.000147 (0.05/340 miRNAs).<br>Model and Bonferroni correction were used in step 1 of the analyses to identify 73 microRNAs and these 73 miRNAs were used for the second analysis.
**miR-20a-5p**	Prospective population-based cohort study [24].	Participants who were enrolled in Framingham Heart Study.	n=1788 participants (4.7% prevalent AF and 8.8% incident AF), mean age 66 ± 9 years, 54.4% women. Median follow-up 8.6 years.	Plasma concentrations associated with incident AF. HR: 0.05, 95% CI: 0.05-0.05 (p=0.05).	Multivariable Cox regression. Model: age, sex, current smoking, diabetes, prevalent heart failure, and myocardial infarction. Bonferroni p=0.000147 (0.05/340 miRNAs).<br>Model and Bonferroni correction were used in step 1 of the analyses to identify 73 microRNAs and these 73 miRNAs were used for the second analysis.
**miR-21**	Cross-sectional Patient-based case-control study [16].	Patients from outpatient clinic (no AF vs. AF no HF vs. no AF with HF and vs. AF, HF).	n=95 patients (34.7% AF), mean age 66.4 ± 1.7 years, 18.9% women.	61% decreased plasma concentrations in AF vs. SR (p=0.041).<br>Plasma concentrations associated with prevalent AF. Beta: -3.7, 95% CI: -7.3 - -0.2 (p=0.041).	Multivariable linear regression. Model: age, sex, coronary artery disease, hypertension, diabetes mellitus, hypercholesterolemia, use of angiotensin converting enzyme inhibitor or AT1-receptor blocker, aldosterone antagonists, and/or statins.
**miR-21-5p**	Prospective patient-based cohort study [21].	Patients enrolled in the miRhythm Study who were evaluated for treatment and that underwent ablation if needed (no AF vs. AF).	n=211 patients (52.6% AF), mean age 58.6 ± 11.9 years, 41.7% women.	2.1-fold decreased plasma concentrations in AF vs. SR (p=<0.05).<br>Plasma concentrations associated with prevalent AF. OR: 0.51, 95% CI: 0.41-0.63 (p=10.0x10^{-10})<br><br>Bonferroni p=0.0006 (0.05/86 miRNAs).	Multivariable logistic regression. Model: age, sex, current smoking, diabetes, prevalent heart failure, and myocardial infarction
**miR-23b-3p**	Prospective population-based cohort study [24].	Participants who were enrolled in Framingham Heart Study.	n=1788 participants (4.7% prevalent AF and 8.8% incident AF), mean age 66 ± 9 years, 54.4% women. Median follow-up 8.6 years.	Plasma concentrations associated with prevalent AF. OR: 1.28, 95% CI: 1.02-1.6 (p=0.032).	Multivariable logistic regression. Model: age, sex, current smoking, diabetes, prevalent heart failure, and myocardial infarction. Bonferroni p=0.000147 (0.05/340 miRNAs).<br>Model and Bonferroni

					correction were used in step 1 of the analyses to identify 73 microRNAs and these 73 miRNAs were used for the second analysis.
**miR-24-3p**	Prospective patient-based cohort study [21].	Patients enrolled in the miRhythm Study who were evaluated for treatment and that underwent ablation if needed (no AF vs. AF).	n=211 patients (52.6% AF), mean age 58.6 ± 11.9 years, 41.7% women.	Plasma concentrations associated with prevalent AF. OR: 0.45, 95% CI: 0.36-0.58 ($p=1.5x10^{-10}$).<br><br>Bonferroni p=0.0006 (0.05/86 miRNAs).	Multivariable logistic regression. Model: age, sex, current smoking, diabetes, prevalent heart failure, and myocardial infarction.
**miR-26a**	Cross-sectional Patient-based case-control study [22].	Patients that underwent ablation for AF were matched with no AF controls.	n=40 patients (75.0% AF), mean age 63.7 ± 2.3 years, 30.0% women.	No difference in plasma concentrations between AF vs. SR (p=NS).	NA.
**miR-26a-5p**	Prospective population-based cohort study [24].	Participants who were enrolled in Framingham Heart Study.	n=1788 participants (4.7% prevalent AF and 8.8% incident AF), mean age 66 ± 9 years, 54.4% women. Median follow-up 8.6 years.	Plasma concentrations associated with prevalent AF. OR: 1.28, 95% CI: 1.07-1.54 (p=0.007).	Multivariable logistic regression. Model: age, sex, current smoking, diabetes, prevalent heart failure, and myocardial infarction. Bonferroni p=0.000147 (0.05/340 miRNAs).<br>Model and Bonferroni correction were used in step 1 of the analyses to identify 73 microRNAs and these 73 miRNAs were used for the second analysis.
**miR-26a-5p**	Prospective population-based cohort study [24].	Participants who were enrolled in Framingham Heart Study.	n=1788 participants (4.7% prevalent AF and 8.8% incident AF), mean age 66 ± 9 years, 54.4% women. Median follow-up 8.6 years.	Plasma concentrations associated with incident AF. HR: 1.16, 95% CI: 1.02-1.32 (p=0.026).	Multivariable Cox regression. Model: age, sex, current smoking, diabetes, prevalent heart failure, and myocardial infarction. Bonferroni p=0.000147 (0.05/340 miRNAs).<br>Model and Bonferroni correction were used in step 1 of the analyses to identify 73 microRNAs and these 73 miRNAs were used for the second analysis.
**miR-27b-3p**	Prospective population-based cohort study [24].	Participants who were enrolled in Framingham Heart Study.	n=1788 participants (4.7% prevalent AF and 8.8% incident AF), mean age 66 ± 9 years, 54.4% women. Median follow-up 8.6 years.	Plasma concentrations associated with prevalent AF. OR: 1.23, 95% CI: 1.01-1.51 (p=0.041).	Multivariable logistic regression. Model: age, sex, current smoking, diabetes, prevalent heart failure, and myocardial infarction. Bonferroni p=0.000147 (0.05/340 miRNAs).<br>Model and Bonferroni correction were used in step 1 of the analyses to identify 73 microRNAs and these 73 miRNAs were used for the second analysis.

**miR-28-5p**	Prospective population-based cohort study [20].	Participants who were enrolled in Framingham Heart Study.	n=2445 participants (6.2% prevalent AF and 6.6% incident AF), mean age 66.3 ± 8.9 years, 56.0% women. Median follow-up 5.4 years.	1.09-fold increased plasma concentrations in AF vs. SR (p=NR).<br>Plasma concentrations associated with prevalent AF.<br>Model 1: OR: 1.15, 95% CI: 1.05-1.26 (p=0.002)<br>Model 2: OR: 1.08, 95% CI: 0.98-1.19 (p=0.14).<br><br>Bonferroni p=2.0×10−4 (0.05/253 miRNAs).	Multivariable logistic regression. Model 1: age, sex, miR-processing.<br>Model 2: age, sex, miR-processing, height, weight, systolic blood pressure, diastolic blood pressure, current smoking, antihypertensive medication use, prevalent heart failure, myocardial infarction, and diabetes mellitus.
**miR-29a-3p**	Prospective patient-based cohort study [21].	Patients enrolled in the miRhythm Study who were evaluated for treatment and that underwent ablation if needed (no AF vs. AF).	n=211 patients (52.6% AF), mean age 58.6 ± 11.9 years, 41.7% women.	Plasma concentrations associated with prevalent AF.<br>OR: 0.47, 95% CI: 0.37-0.60 (p=$2.5x10^{-10}$)<br><br>Bonferroni p=0.0006 (0.05/86 miRNAs).	Multivariable logistic regression. Model: age, sex, current smoking, diabetes, prevalent heart failure, and myocardial infarction.
**miR-29b**	Cross-sectional Patient-based case-control study [16].	Patients from outpatient clinic (no AF vs. AF no HF vs. no AF with HF and vs. AF, HF).	n=95 patients (34.7% AF), mean age 66.4 ± 1.7 years, 18.9% women.	54% decreased plasma concentrations in AF vs. SR (p=<0.001).<br>Plasma concentrations associated with prevalent AF.<br>Beta: -10.5, 95% CI: -17.9 - -3.1 (p=0.007).	Multivariable linear regression. Model: age, sex, coronary artery disease, hypertension, diabetes mellitus, hypercholesterolemia, use of angiotensin converting enzyme inhibitor or AT1-receptor blocker, aldosterone antagonists, and/or statins.
**miR-29b-2-5p**	Prospective population-based cohort study [20].	Participants who were enrolled in Framingham Heart Study.	n=2445 participants (6.2% prevalent AF and 6.6% incident AF), mean age 66.3 ± 8.9 years, 56.0% women. Median follow-up 5.4 years.	0.99-fold increased plasma concentrations in AF vs. SR (p=NR).<br>Plasma concentrations associated with incident AF.<br>Model 1: OR: 0.84, 95% CI: 0.73-0.97 (p=0.02)<br>Model 2: OR: 0.83, 95% CI: 0.72-0.97 (p=0.016).<br><br>Bonferroni p=$2.0×10^{-4}$ (0.05/253 miRNAs).	Multivariable logistic regression. Model 1: age, sex, miR-processing.<br>Model 2: age, sex, miR-processing, height, weight, systolic blood pressure, diastolic blood pressure, current smoking, antihypertensive medication use, prevalent heart failure, myocardial infarction, and diabetes mellitus.
**miR-30a-5p**	Prospective population-based cohort study [24].	Participants who were enrolled in Framingham Heart Study.	n=1788 participants (4.7% prevalent AF and 8.8% incident AF), mean age 66 ± 9 years, 54.4% women. Median follow-up 8.6 years.	Plasma concentrations associated with prevalent AF.<br>OR: 1.25, 95% CI: 1.05-1.49 (p=0.013).	Multivariable logistic regression. Model: age, sex, current smoking, diabetes, prevalent heart failure, and myocardial infarction.<br>Bonferroni p=0.000147 (0.05/340 miRNAs).<br>Model and Bonferroni correction were used in step 1 of the analyses to identify 73 microRNAs and these 73 miRNAs were used for the second analysis.
**miR-30c-5p**	Prospective patient-based cohort study [21].	Patients enrolled in the miRhythm Study who were evaluated for treatment and that underwent ablation	n=211 patients (52.6% AF), mean age 58.6 ± 11.9 years, 41.7% women.	Plasma concentrations associated with prevalent AF.<br>OR: 0.41, 95% CI: 0.32-0.54 (p=$8.0x10^{-11}$).<br><br>Bonferroni p=0.0006 (0.05/86 miRNAs).	Multivariable logistic regression. Model: age, sex, current smoking, diabetes, prevalent heart failure, and myocardial infarction.

		if needed (no AF vs. AF).			
**miR-31-3p**	Prospective population-based cohort study [20].	Participants who were enrolled in Framingham Heart Study.	n=2445 participants (6.2% prevalent AF and 6.6% incident AF), mean age 66.3 ± 8.9 years, 56.0% women. Median follow-up 5.4 years.	0.98-fold increased plasma concentrations in AF vs. SR (p=NR).<br>Plasma concentrations associated with prevalent AF.<br>Model 1: OR: 0.69, 95% CI: 0.54-0.89 (p=0.004)<br>Model 2: OR: 0.69, 95% CI: 0.54-0.90 (p=0.005).<br><br>Bonferroni $p=2.0\times10^{-4}$ (0.05/253 miRNAs).	Multivariable logistic regression. Model 1: age, sex, miR-processing.<br>Model 2: age, sex, miR-processing, height, weight, systolic blood pressure, diastolic blood pressure, current smoking, antihypertensive medication use, prevalent heart failure, myocardial infarction, and diabetes mellitus.
**miR-93-5p**	Prospective population-based cohort study [24].	Participants who were enrolled in Framingham Heart Study.	n=1788 participants (4.7% prevalent AF and 8.8% incident AF), mean age 66 ± 9 years, 54.4% women. Median follow-up 8.6 years.	Plasma concentrations associated with prevalent AF. OR: 1.28, 95% CI: 1.06-1.55 (p=0.011).	Multivariable logistic regression. Model: age, sex, current smoking, diabetes, prevalent heart failure, and myocardial infarction.<br>Bonferroni p=0.000147 (0.05/340 miRNAs).<br>Model and Bonferroni correction were used in step 1 of the analyses to identify 73 microRNAs and these 73 miRNAs were used for the second analysis.
**miR-99b-5p**	Prospective patient-based cohort study [21].	Patients enrolled in the miRhythm Study who were evaluated for treatment and that underwent ablation if needed (no AF vs. AF).	n=211 patients (52.6% AF), mean age 58.6 ± 11.9 years, 41.7% women.	Plasma concentrations associated with prevalent AF. OR: 0.64, 95% CI: 0.53-0.77 ($p=1.980x10^{-6}$).<br><br>Bonferroni p=0.0006 (0.05/86 miRNAs).	Multivariable logistic regression. Model: age, sex, current smoking, diabetes, prevalent heart failure, and myocardial infarction.
**miR-99b-5p**	Prospective population-based cohort study [20].	Participants who were enrolled in Framingham Heart Study.	n=2445 participants (6.2% prevalent AF and 6.6% incident AF), mean age 66.3 ± 8.9 years, 56.0% women. Median follow-up 5.4 years.	1.07-fold increased plasma concentrations in AF vs. SR (p=NR).<br>Plasma concentrations associated with prevalent AF.<br>Model 1: OR: 1.12, 95% CI: 1.04-1.20 (p=0.003)<br>Model 2: OR: 1.05, 95% CI: 0.96-1.16 (p=0.27).<br><br>Bonferroni $p=2.0\times10^{-4}$ (0.05/253 miRNAs).	Multivariable logistic regression. Model 1: age, sex, miR-processing.<br>Model 2: age, sex, miR-processing, height, weight, systolic blood pressure, diastolic blood pressure, current smoking, antihypertensive medication use, prevalent heart failure, myocardial infarction, and diabetes mellitus.
**miR-100-5p**	Prospective patient-based cohort study [21].	Patients enrolled in the miRhythm Study who were evaluated for treatment and that underwent ablation if needed (no AF vs. AF).	n=211 patients (52.6% AF), mean age 58.6 ± 11.9 years, 41.7% women.	Plasma concentrations associated with prevalent AF. OR: 0.42, 95% CI: 0.33-0.54 ($p=4.5x10^{-12}$)<br><br>Bonferroni p=0.0006 (0.05/86 miRNAs).	Multivariable logistic regression. Model: age, sex, current smoking, diabetes, prevalent heart failure, and myocardial infarction.
**miR-106b-5p**	Prospective population-based cohort study [24].	Participants who were enrolled in Framingham Heart Study.	n=1788 participants (4.7% prevalent AF and 8.8% incident AF), mean age 66 ± 9 years, 54.4%	Plasma concentrations associated with prevalent AF. OR: 1.36, 95% CI: 1.13-1.63 (p=0.001).	Multivariable logistic regression. Model: age, sex, current smoking, diabetes, prevalent heart failure, and myocardial infarction.

			women. Median follow-up 8.6 years.		Bonferroni p=0.000147 (0.05/340 miRNAs).<br>Model and Bonferroni correction were used in step 1 of the analyses to identify 73 microRNAs and these 73 miRNAs were used for the second analysis.
**miR-106b-5p**	Prospective population-based cohort study [24].	Participants who were enrolled in Framingham Heart Study.	n=1788 participants (4.7% prevalent AF and 8.8% incident AF), mean age 66 ± 9 years, 54.4% women. Median follow-up 8.6 years.	Plasma concentrations associated with incident AF. HR: 1.16, 95% CI: 1.02-1.33 (p=0.029).	Multivariable Cox regression. Model: age, sex, current smoking, diabetes, prevalent heart failure, and myocardial infarction. Bonferroni p=0.000147 (0.05/340 miRNAs).<br>Model and Bonferroni correction were used in step 1 of the analyses to identify 73 microRNAs and these 73 miRNAs were used for the second analysis.
**miR-122-5p**	Prospective patient-based cohort study [21].	Patients enrolled in the miRhythm Study who were evaluated for treatment and that underwent ablation if needed (no AF vs. AF).	n=211 patients (52.6% AF), mean age 58.6 ± 11.9 years, 41.7% women.	Plasma concentrations associated with prevalent AF. OR: 0.55, 95% CI: 0.46-0.67 ($p=6.3x10^{-10}$).<br><br>Bonferroni p=0.0006 (0.05/86 miRNAs).	Multivariable logistic regression. Model: age, sex, current smoking, diabetes, prevalent heart failure, and myocardial infarction.
**miR-125a-5p**	Prospective patient-based cohort study [21].	Patients enrolled in the miRhythm Study who were evaluated for treatment and that underwent ablation if needed (no AF vs. AF).	n=211 patients (52.6% AF), mean age 58.6 ± 11.9 years, 41.7% women.	Plasma concentrations associated with prevalent AF. OR: 0.47, 95% CI: 0.38-0.58 ($p=5.0x10^{-12}$)<br><br>Bonferroni p=0.0006 (0.05/86 miRNAs).	Multivariable logistic regression. Model: age, sex, current smoking, diabetes, prevalent heart failure, and myocardial infarction.
**miR-125b-5p**	Prospective patient-based cohort study [21].	Patients enrolled in the miRhythm Study who were evaluated for treatment and that underwent ablation if needed (no AF vs. AF).	n=211 patients (52.6% AF), mean age 58.6 ± 11.9 years, 41.7% women.	Plasma concentrations associated with prevalent AF. OR: 0.50, 95% CI: 0.41-0.62 ($p=5.7x10^{-11}$).<br><br>Bonferroni p=0.0006 (0.05/86 miRNAs).	Multivariable logistic regression. Model: age, sex, current smoking, diabetes, prevalent heart failure, and myocardial infarction
**miR-126**	Cross-sectional Patient-based case-control study [25].	Patients admitted to the department of Cardiology (no AF vs. AF no HF vs. no AF with HF and vs. AF, HF).	n=135 patients (52.6% AF), mean age 63.9 ± 15.1 years, 48.1% women.	3.4-fold decreased plasma concentrations in AF vs. SR (p=<0.01).	NA.
**miR-126-3p**	Prospective patient-based cohort study [21].	Patients enrolled in the miRhythm Study who were evaluated for treatment and that underwent ablation if needed (no AF vs. AF).	n=211 patients (52.6% AF), mean age 58.6 ± 11.9 years, 41.7% women.	Plasma concentrations associated with prevalent AF. OR: 0.45, 95% CI: 0.35-0.57 ($p=8.0x10^{-11}$).<br><br>Bonferroni p=0.0006 (0.05/86 miRNAs).	Multivariable logistic regression. Model: age, sex, current smoking, diabetes, prevalent heart failure, and myocardial infarction.

**miR-126-5p**	Prospective population-based cohort study [24].	Participants who were enrolled in Framingham Heart Study.	n=1788 participants (4.7% prevalent AF and 8.8% incident AF), mean age 66 ± 9 years, 54.4% women. Median follow-up 8.6 years.	Plasma concentrations associated with prevalent AF. OR: 1.32, 95% CI: 1.1-1.58 (p=0.003).	Multivariable logistic regression. Model: age, sex, current smoking, diabetes, prevalent heart failure, and myocardial infarction. Bonferroni p=0.000147 (0.05/340 miRNAs). Model and Bonferroni correction were used in step 1 of the analyses to identify 73 microRNAs and these 73 miRNAs were used for the second analysis.
**miR-133a**	Prospective patient-based cohort study [23].	Patients that underwent on-pump CABG without history of AF were monitored for POAF.	n=42 patients (16.7% POAF), mean age 65.0 ± 1.3 years, 26.2% women. Follow-up to 7 days after surgery by continuous ECG monitoring.	No difference in plasma concentrations between POAF vs. SR (p=NS).	NA.
**miR-133a**	Cross-sectional Patient-based case-control study [16].	Patients from outpatient clinic (no AF vs. AF no HF vs. no AF with HF and vs. AF, HF).	n=95 patients (34.7% AF), mean age 66.4 ± 1.7 years, 18.9% women.	No difference in plasma concentrations between AF vs. SR (p=NS). Plasma concentrations were not associated with prevalent AF. Beta: -0.86, 95% CI: -1.88 - 0.15 (p=0.09).	Multivariable linear regression. Model: age, sex, coronary artery disease, hypertension, diabetes mellitus, hypercholesterolemia, use of angiotensin converting enzyme inhibitor or AT1-receptor blocker, aldosterone antagonists, and/or statins.
**miR-133a**	Cross-sectional Patient-based case-control study [22].	Patients that underwent ablation for AF were matched with WPW patients as controls.	n=40 patients (75.0% AF), mean age 63.7 ± 2.3 years, 30.0% women.	No difference in plasma concentrations between AF vs. SR (p=NS).	NA.
**miR-134**	Prospective population-based cohort study [20].	Participants who were enrolled in Framingham Heart Study.	n=2445 participants (6.2% prevalent AF and 6.6% incident AF), mean age 66.3 ± 8.9 years, 56.0% women. Median follow-up 5.4 years.	0.97-fold increased plasma concentrations in AF vs. SR (p=NR).<br>Plasma concentrations associated with incident AF.<br>Model 1: OR: 0.86, 95% CI: 0.76-0.97 (p=0.013).<br>Model 2: OR: 0.85, 95% CI: 0.75-0.96 (p=0.009).<br><br>Bonferroni $p=2.0\times10^{-4}$ (0.05/253 miRNAs).	Multivariable logistic regression. Model 1: age, sex, miR-processing.<br>Model 2: age, sex, miR-processing, height, weight, systolic blood pressure, diastolic blood pressure, current smoking, antihypertensive medication use, prevalent heart failure, myocardial infarction, and diabetes mellitus.
**miR-140-3p**	Prospective population-based cohort study [24].	Participants who were enrolled in Framingham Heart Study.	n=1788 participants (4.7% prevalent AF and 8.8% incident AF), mean age 66 ± 9 years, 54.4% women. Median follow-up 8.6 years.	Plasma concentrations associated with prevalent AF. OR: 1.31, 95% CI: 1.05-1.63 (p=0.015).	Multivariable logistic regression. Model: age, sex, current smoking, diabetes, prevalent heart failure, and myocardial infarction. Bonferroni p=0.000147 (0.05/340 miRNAs). Model and Bonferroni correction were used in step 1 of the analyses to identify 73 microRNAs and these 73 miRNAs were used for the second analysis.

**miR-146a-5p**	Prospective patient-based cohort study [21].	Patients enrolled in the miRhythm Study who were evaluated for treatment and that underwent ablation if needed (no AF vs. AF).	n=211 patients (52.6% AF), mean age 58.6 ± 11.9 years, 41.7% women.	Plasma concentrations associated with prevalent AF. OR: 0.38, 95% CI: 0.29-0.50 ($p=1.3x10^{-11}$).<br><br>Bonferroni p=0.0006 (0.05/86 miRNAs).	Multivariable logistic regression. Model: age, sex, current smoking, diabetes, prevalent heart failure, and myocardial infarction.
**miR-148b-3p**	Prospective patient-based cohort study [21].	Patients enrolled in the miRhythm Study who were evaluated for treatment and that underwent ablation if needed (no AF vs. AF).	n=211 patients (52.6% AF), mean age 58.6 ± 11.9 years, 41.7% women.	Plasma concentrations associated with prevalent AF. OR: 0.46, 95% CI: 0.36-0.59 ($p=5.9x10^{-10}$).<br><br>Bonferroni p=0.0006 (0.05/86 miRNAs).	Multivariable logistic regression. Model: age, sex, current smoking, diabetes, prevalent heart failure, and myocardial infarction.
**miR-150-5p**	Prospective patient-based cohort study [21].	Patients enrolled in the miRhythm study who were evaluated for treatment and that underwent ablation if needed (no AF vs. AF).	n=211 patients (52.6% AF), mean age 58.6 ± 11.9 years, 41.7% women.	2.3-fold decreased plasma concentrations in AF vs. SR (p=<0.05). Plasma concentrations associated with prevalent AF. OR: 0.51, 95% CI: 0.41-0.63 ($p=1.7x10^{-10}$).<br><br>Bonferroni p=0.0006 (0.05/86 miRNAs).	Multivariable logistic regression. Model: age, sex, current smoking, diabetes, prevalent heart failure, and myocardial infarction
**miR-150-5p**	Prospective population-based cohort study [20].	Participants who were enrolled in Framingham Heart Study.	n=2445 participants (6.2% prevalent AF and 6.6% incident AF), mean age 66.3 ± 8.9 years, 56.0% women. Median follow-up 5.4 years.	1.10-fold increased plasma concentrations in AF vs. SR (p=NR).<br>Plasma concentrations associated with prevalent AF.<br>Model 1: OR: 1.29, 95% CI: 1.11-1.50 (p=0.001).<br>Model 2: OR: 1.24, 95% CI: 1.04-1.49 (p=0.018).<br><br>Bonferroni $p=2.0\times10^{-4}$ (0.05/253 miRNAs).	Multivariable logistic regression. Model 1: age, sex, miR-processing.<br>Model 2: age, sex, miR-processing, height, weight, systolic blood pressure, diastolic blood pressure, current smoking, antihypertensive medication use, prevalent heart failure, myocardial infarction, and diabetes mellitus.
**miR-150-5p**	Prospective population-based cohort study [24].	Participants who were enrolled in Framingham Heart Study.	n=1788 participants (4.7% prevalent AF and 8.8% incident AF), mean age 66 ± 9 years, 54.4% women. Median follow-up 8.6 years.	Plasma concentrations associated with prevalent AF. OR: 1.21, 95% CI: 1.04-1.41 (p=0.013).	Multivariable logistic regression. Model: age, sex, current smoking, diabetes, prevalent heart failure, and myocardial infarction.<br>Bonferroni p=0.000147 (0.05/340 miRNAs).<br>Model and Bonferroni correction were used in step 1 of the analyses to identify 73 microRNAs and these 73 miRNAs were used for the second analysis.

**miR-151a-3p**	Prospective population-based cohort study [20].	Participants who were enrolled in Framingham Heart Study.	n=2445 participants (6.2% prevalent AF and 6.6% incident AF), mean age 66.3 ± 8.9 years, 56.0% women. Median follow-up 5.4 years.	0.99-fold increased plasma concentrations in AF vs. SR (p=NR).<br>Plasma concentrations associated with incident AF.<br>Model 1: OR: 0.87, 95% CI: 0.78-0.98 (p=0.019).<br>Model 2: OR: 0.88, 95% CI: 0.78-0.99 (p=0.031).<br><br>Bonferroni p=2.0×10^{-4} (0.05/253 miRNAs).	Multivariable logistic regression. Model 1: age, sex, miR-processing.<br>Model 2: age, sex, miR-processing, height, weight, systolic blood pressure, diastolic blood pressure, current smoking, antihypertensive medication use, prevalent heart failure, myocardial infarction, and diabetes mellitus.
**miR-152**	Prospective population-based cohort study [20].	Participants who were enrolled in Framingham Heart Study.	n=2445 participants (6.2% prevalent AF and 6.6% incident AF), mean age 66.3 ± 8.9 years, 56.0% women. Median follow-up 5.4 years.	0.97-fold increased plasma concentrations in AF vs. SR (p=NR).<br>Plasma concentrations associated with incident AF.<br>Model 1: OR: 0.87, 95% CI: 0.79-0.95 (p=0.001).<br>Model 2: OR: 0.89, 95% CI: 0.81-0.98 (p=0.015).<br><br>Bonferroni p=2.0×10^{-4} (0.05/253 miRNAs).	Multivariable logistic regression. Model 1: age, sex, miR-processing.<br>Model 2: age, sex, miR-processing, height, weight, systolic blood pressure, diastolic blood pressure, current smoking, antihypertensive medication use, prevalent heart failure, myocardial infarction, and diabetes mellitus.
**miR-182-5p**	Prospective population-based cohort study [20].	Participants who were enrolled in Framingham Heart Study.	n=2445 participants (6.2% prevalent AF and 6.6% incident AF), mean age 66.3 ± 8.9 years, 56.0% women. Median follow-up 5.4 years.	0.94-fold increased plasma concentrations in AF vs. SR (p=NR).<br>Plasma concentrations associated with prevalent AF.<br>Model 1: OR: 0.91, 95% CI: 0.85-0.97 (p=0.003).<br>Model 2: OR: 0.93, 95% CI: 0.87-1.00 (p=0.04).<br><br>Bonferroni p=2.0×10^{-4} (0.05/253 miRNAs).	Multivariable logistic regression. Model 1: age, sex, miR-processing.<br>Model 2: age, sex, miR-processing, height, weight, systolic blood pressure, diastolic blood pressure, current smoking, antihypertensive medication use, prevalent heart failure, myocardial infarction, and diabetes mellitus.
**miR-186-5p**	Prospective population-based cohort study [24].	Participants who were enrolled in Framingham Heart Study.	n=1788 participants (4.7% prevalent AF and 8.8% incident AF), mean age 66 ± 9 years, 54.4% women. Median follow-up 8.6 years.	Plasma concentrations associated with prevalent AF.<br>OR: 1.28, 95% CI: 1.02-1.61 (p=0.340).	Multivariable logistic regression. Model: age, sex, current smoking, diabetes, prevalent heart failure, and myocardial infarction.<br>Bonferroni p=0.000147 (0.05/340 miRNAs).<br>Model and Bonferroni correction were used in step 1 of the analyses to identify 73 microRNAs and these 73 miRNAs were used for the second analysis.

**miR-193a-5p**	Prospective population-based cohort study [20].	Participants who were enrolled in Framingham Heart Study.	n=2445 participants (6.2% prevalent AF and 6.6% incident AF), mean age 66.3 ± 8.9 years, 56.0% women. Median follow-up 5.4 years.	0.98-fold increased plasma concentrations in AF vs. SR (p=NR).<br>Plasma concentrations associated with incident AF.<br>Model 1: OR: 0.87, 95% CI: 0.77-0.98 (p=0.018).<br>Model 2: OR: 0.87, 95% CI: 0.77-0.98 (p=0.024).<br><br>Bonferroni $p=2.0\times10^{-4}$ (0.05/253 miRNAs).	Multivariable logistic regression. Model 1: age, sex, miR-processing.<br>Model 2: age, sex, miR-processing, height, weight, systolic blood pressure, diastolic blood pressure, current smoking, antihypertensive medication use, prevalent heart failure, myocardial infarction, and diabetes mellitus.
**miR-196b-5p**	Prospective population-based cohort study [20].	Participants who were enrolled in Framingham Heart Study.	n=2445 participants (6.2% prevalent AF and 6.6% incident AF), mean age 66.3 ± 8.9 years, 56.0% women. Median follow-up 5.4 years.	0.97-fold increased plasma concentrations in AF vs. SR (p=NR).<br>Plasma concentrations associated with prevalent AF.<br>Model 1: OR: 0.84, 95% CI: 0.75-0.95 (p=0.005).<br>Model 2: OR: 0.83, 95% CI: 0.73-0.94 (p=0.004).<br><br>Bonferroni $p=2.0\times10^{-4}$ (0.05/253 miRNAs).	Multivariable logistic regression. Model 1: age, sex, miR-processing.<br>Model 2: age, sex, miR-processing, height, weight, systolic blood pressure, diastolic blood pressure, current smoking, antihypertensive medication use, prevalent heart failure, myocardial infarction, and diabetes mellitus.
**miR-199a-3p**	Prospective population-based cohort study [24].	Participants who were enrolled in Framingham Heart Study.	n=1788 participants (4.7% prevalent AF and 8.8% incident AF), mean age 66 ± 9 years, 54.4% women. Median follow-up 8.6 years.	Plasma concentrations associated with prevalent AF.<br>OR: 1.26, 95% CI: 1.04-1.53 (p=0.019).	Multivariable logistic regression. Model: age, sex, current smoking, diabetes, prevalent heart failure, and myocardial infarction.<br>Bonferroni p=0.000147 (0.05/340 miRNAs).<br>Model and Bonferroni correction were used in step 1 of the analyses to identify 73 microRNAs and these 73 miRNAs were used for the second analysis.
**miR-200c-3p**	Prospective population-based cohort study [20].	Participants who were enrolled in Framingham Heart Study.	n=2445 participants (6.2% prevalent AF and 6.6% incident AF), mean age 66.3 ± 8.9 years, 56.0% women. Median follow-up 5.4 years.	0.96-fold increased plasma concentrations in AF vs. SR (p=NR).<br>Plasma concentrations associated with incident AF.<br>Model 1: OR: 0.88, 95% CI: 0.80-0.98 (p=0.017).<br>Model 2: OR: 0.89, 95% CI: 0.80-0.98 (p=0.022).<br><br>Bonferroni $p=2.0\times10^{-4}$ (0.05/253 miRNAs).	Multivariable logistic regression. Model 1: age, sex, miR-processing.<br>Model 2: age, sex, miR-processing, height, weight, systolic blood pressure, diastolic blood pressure, current smoking, antihypertensive medication use, prevalent heart failure, myocardial infarction, and diabetes mellitus.

**miR-221-3p**	Prospective patient-based cohort study [21].	Patients enrolled in the miRhythm Study who were evaluated for treatment and that underwent ablation if needed (no AF vs. AF).	n=211 patients (52.6% AF), mean age 58.6 ± 11.9 years, 41.7% women.	Plasma concentrations associated with prevalent AF. OR: 0.49, 95% CI: 0.39-0.61 ($p=2.3x10^{-10}$).<br><br>Bonferroni p=0.0006 (0.05/86 miRNAs).	Multivariable logistic regression. Model: age, sex, current smoking, diabetes, prevalent heart failure, and myocardial infarction.
**miR-221-3p**	Prospective population-based cohort study [20].	Participants who were enrolled in Framingham Heart Study.	n=2445 participants (6.2% prevalent AF and 6.6% incident AF), mean age 66.3 ± 8.9 years, 56.0% women. Median follow-up 5.4 years.	0.99-fold increased plasma concentrations in AF vs. SR (p=NR).<br>Plasma concentrations associated with incident AF.<br>Model 1: OR: 0.86, 95% CI: 0.76-0.96 (p=0.01).<br>Model 2: OR: 0.85, 95% CI: 0.76-0.97 (p=0.012).<br><br>Bonferroni $p=2.0\times10^{-4}$ (0.05/253 miRNAs).	Multivariable logistic regression. Model 1: age, sex, miR-processing.<br>Model 2: age, sex, miR-processing, height, weight, systolic blood pressure, diastolic blood pressure, current smoking, antihypertensive medication use, prevalent heart failure, myocardial infarction, and diabetes mellitus.
**miR-223-3p**	Prospective patient-based cohort study [21].	Patients enrolled in the miRhythm Study who were evaluated for treatment and that underwent ablation if needed (no AF vs. AF).	n=211 patients (52.6% AF), mean age 58.6 ± 11.9 years, 41.7% women.	Plasma concentrations associated with prevalent AF. OR: 0.48, 95% CI: 0.39-0.60 ($p=5.9x10^{-11}$).<br><br>Bonferroni p=0.0006 (0.05/86 miRNAs).	Multivariable logistic regression. Model: age, sex, current smoking, diabetes, prevalent heart failure, and myocardial infarction.
**miR-324-3p**	Prospective population-based cohort study [24].	Participants who were enrolled in Framingham Heart Study.	n=1788 participants (4.7% prevalent AF and 8.8% incident AF), mean age 66 ± 9 years, 54.4% women. Median follow-up 8.6 years.	Plasma concentrations associated with incident AF. HR: 1.19, 95% CI: 1.03-1.36 (p=0.016).	Multivariable Cox regression. Model: age, sex, current smoking, diabetes, prevalent heart failure, and myocardial infarction. Bonferroni p=0.000147 (0.05/340 miRNAs).<br>Model and Bonferroni correction were used in step 1 of the analyses to identify 73 microRNAs and these 73 miRNAs were used for the second analysis.
**miR-328**	Cross-sectional Patient-based case-control study [22].	Patients that underwent ablation for AF were matched with WPW patients as controls.	n=40 patients (75.0% AF), mean age 63.7 ± 2.3 years, 30.0% women.	No difference in plasma concentrations between AF vs. SR (p=NS).	NA.
**miR-328**	Cross-sectional patient-based case-control study [19].	Patients that underwent ablation for AF were age-sex matched with no AF controls.	n=200 patients (50.0% AF), mean age 53.1 ± 12.2 years, 28.0% women.	37% decreased pre-operative plasma concentrations in AF vs. SR (p=0.217).	NA.
**miR-328**	Prospective population-based cohort study [20].	Participants who were enrolled in Framingham Heart Study.	n=2445 participants (6.2% prevalent AF and 6.6% incident AF), mean age 66.3 ± 8.9 years,	1.13-fold increased plasma concentrations in AF vs. SR (p=NR).<br>Plasma concentrations associated with prevalent AF.	Multivariable logistic regression. Model 1: age, sex, miR-processing.<br>Model 2: age, sex, miR-processing, height, weight,

			56.0% women. Median follow-up 5.4 years.	Model 1: OR: 1.21, 95% CI: 1.09-1.33 (p=0.00018).<br>Model 2: OR: 1.14, 95% CI: 1.02-1.28 (p=0.017).<br><br>Bonferroni $p=2.0\times10^{-4}$ (0.05/253 miRNAs).	systolic blood pressure, diastolic blood pressure, current smoking, antihypertensive medication use, prevalent heart failure, myocardial infarction, and diabetes mellitus.
**miR-331-3p**	Prospective population-based cohort study [20].	Participants who were enrolled in Framingham Heart Study.	n=2445 participants (6.2% prevalent AF and 6.6% incident AF), mean age 66.3 ± 8.9 years, 56.0% women. Median follow-up 5.4 years.	1.09-fold increased plasma concentrations in AF vs. SR (p=NR).<br>Plasma concentrations associated with prevalent AF.<br>Model 1: OR: 1.18, 95% CI: 1.06-1.31 (p=0.002).<br>Model 2: OR: 1.14, 95% CI: 1.01-1.28 (p=0.035).<br><br>Bonferroni $p=2.0\times10^{-4}$ (0.05/253 miRNAs).	Multivariable logistic regression. Model 1: age, sex, miR-processing.<br>Model 2: age, sex, miR-processing, height, weight, systolic blood pressure, diastolic blood pressure, current smoking, antihypertensive medication use, prevalent heart failure, myocardial infarction, and diabetes mellitus.
**miR-339-5p**	Prospective population-based cohort study [20].	Participants who were enrolled in Framingham Heart Study.	n=2445 participants (6.2% prevalent AF and 6.6% incident AF), mean age 66.3 ± 8.9 years, 56.0% women. Median follow-up 5.4 years.	1.04-fold increased plasma concentrations in AF vs. SR (p=NR).<br>Plasma concentrations associated with prevalent AF.<br>Model 1: OR: 1.13, 95% CI: 1.04-1.23 (p=0.006).<br>Model 2: OR: 1.10, 95% CI: 1.00-1.21 (p=0.05).<br><br>Bonferroni $p=2.0\times10^{-4}$ (0.05/253 miRNAs).	Multivariable logistic regression. Model 1: age, sex, miR-processing.<br>Model 2: age, sex, miR-processing, height, weight, systolic blood pressure, diastolic blood pressure, current smoking, antihypertensive medication use, prevalent heart failure, myocardial infarction, and diabetes mellitus.
**miR-342-3p**	Prospective patient-based cohort study [21].	Patients enrolled in the miRhythm Study who were evaluated for treatment and that underwent ablation if needed (no AF vs. AF).	n=211 patients (52.6% AF), mean age 58.6 ± 11.9 years, 41.7% women.	Plasma concentrations associated with prevalent AF.<br>OR: 0.31, 95% CI: 0.23-0.43 ($p=2.4\times10^{-12}$)<br><br>Bonferroni p=0.0006 (0.05/86 miRNAs).	Multivariable logistic regression. Model: age, sex, current smoking, diabetes, prevalent heart failure, and myocardial infarction.
**miR-363-3p**	Prospective population-based cohort study [24].	Participants who were enrolled in Framingham Heart Study.	n=1788 participants (4.7% prevalent AF and 8.8% incident AF), mean age 66 ± 9 years, 54.4% women. Median follow-up 8.6 years.	Plasma concentrations associated with incident AF.<br>HR: 1.17, 95% CI: 1.01-1.36 (p=0.043).	Multivariable Cox regression. Model: age, sex, current smoking, diabetes, prevalent heart failure, and myocardial infarction. Bonferroni p=0.000147 (0.05/340 miRNAs).<br>Model and Bonferroni correction were used in step 1 of the analyses to identify 73 microRNAs and these 73 miRNAs were used for the second analysis.
**miR-375**	Prospective patient-based cohort study [21].	Patients enrolled in the miRhythm Study who were evaluated for treatment and that underwent ablation if needed (no AF vs. AF).	n=211 patients (52.6% AF), mean age 58.6 ± 11.9 years, 41.7% women.	Plasma concentrations associated with prevalent AF.<br>Model: OR: 0.36, 95% CI: 0.26-0.50 ($p=2.7\times10^{-10}$).<br><br>Bonferroni p=0.0006 (0.05/86 miRNAs).	Multivariable logistic regression. Model: age, sex, current smoking, diabetes, prevalent heart failure, and myocardial infarction.

**miR-375**	Prospective population-based cohort study [20].	Participants who were enrolled in Framingham Heart Study.	n=2445 participants (6.2% prevalent AF and 6.6% incident AF), mean age 66.3 ± 8.9 years, 56.0% women. Median follow-up 5.4 years.	0.99-fold increased plasma concentrations in AF vs. SR (p=NR).<br>Plasma concentrations associated with incident AF.<br>Model 1: OR: 0.83, 95% CI: 0.72-0.96 (p=0.001).<br>Model 2: OR: 0.82, 95% CI: 0.71-0.95 (p=0.007).<br><br>Bonferroni p=2.0×10^{-4} (0.05/253 miRNAs).	Multivariable logistic regression. Model 1: age, sex, miR-processing.<br>Model 2: age, sex, miR-processing, height, weight, systolic blood pressure, diastolic blood pressure, current smoking, antihypertensive medication use, prevalent heart failure, myocardial infarction, and diabetes mellitus.
**miR-409-3p**	Cross-sectional patient-based case-control study [19].	Patients that underwent ablation for AF were age-sex matched with no AF controls.	n=200 patients (50.0% AF), mean age 53.1 ± 12.2 years, 28.0% women.	54% decreased pre-operative plasma concentrations in AF vs. SR (p=<0.001).<br>Plasma concentrations associated with prevalent AF. OR: 1.50, 95% CI: 1.02-2.22 (p=0.040).	Multivariable logistic regression. Model: age, gender, hypertension.
**miR-411-5p**	Prospective patient-based cohort study [21].	Patients enrolled in the miRhythm Study who were evaluated for treatment and that underwent ablation if needed (no AF vs. AF).	n=211 patients (52.6% AF), mean age 58.6 ± 11.9 years, 41.7% women.	Plasma concentrations associated with prevalent AF. OR: 0.42, 95% CI: 0.31-0.56 (p=4.3x10^{-9}).<br><br>Bonferroni p=0.0006 (0.05/86 miRNAs).	Multivariable logistic regression. Model: age, sex, current smoking, diabetes, prevalent heart failure, and myocardial infarction.
**miR-432**	Cross-sectional patient-based case-control study [19].	Patients that underwent ablation for AF were age-sex matched with no AF controls.	n=200 patients (50.0% AF), mean age 53.1 ± 12.2 years, 28.0% women.	68% decreased pre-operative plasma concentrations in AF vs. SR (p=<0.001).<br>Plasma concentrations associated with prevalent AF. OR: 1.63, 95% CI: 1.09-2.43 (p=0.018).	Multivariable logistic regression. Model: age, gender, hypertension.
**miR-483-5p**	Prospective patient-based cohort study [17].	Patients that underwent on-pump CABG without history of AF were monitored for POAF.	n=34 patients (38.2% POAF), mean age 61.5 ± 11.8 years, 29.4% women. Follow-up from time of surgery to hospital discharge by continuous ECG monitoring.	Increased pre-operative plasma concentrations in AF vs. SR (p=0.0137).	NA.
**miR-484**	Prospective population-based cohort study [24].	Participants who were enrolled in Framingham Heart Study.	n=1788 participants (4.7% prevalent AF and 8.8% incident AF), mean age 66 ± 9 years, 54.4% women. Median follow-up 8.6 years.	Plasma concentrations associated with prevalent AF. OR: 1.3, 95% CI: 1.07-1.58 (p=0.007).	Multivariable logistic regression. Model: age, sex, current smoking, diabetes, prevalent heart failure, and myocardial infarction. Bonferroni p=0.000147 (0.05/340 miRNAs).<br>Model and Bonferroni correction were used in step 1 of the analyses to identify 73 microRNAs and these 73 miRNAs were used for the second analysis.
**miR-484**	Prospective population-based cohort study [24].	Participants who were enrolled in Framingham Heart Study.	n=1788 participants (4.7% prevalent AF and 8.8% incident AF), mean age 66 ± 9 years, 54.4% women. Median follow-up 8.6 years.	Plasma concentrations associated with incident AF. HR: 1.15, 95% CI: 1-1.32 (p=0.049).	Multivariable Cox regression. Model: age, sex, current smoking, diabetes, prevalent heart failure, and myocardial infarction. Bonferroni p=0.000147 (0.05/340 miRNAs).

					Model and Bonferroni correction were used in step 1 of the analyses to identify 73 microRNAs and these 73 miRNAs were used for the second analysis.
**miR-590**	Cross-sectional Patient-based case-control study [22].	Patients that underwent ablation for AF were matched with WPW patients as controls.	n=40 patients (75.0% AF), mean age 63.7 ± 2.3 years, 30.0% women.	No difference in plasma concentrations between AF vs. SR (p=NS).	NA.
**miR-720**	Prospective population-based cohort study [20].	Participants who were enrolled in Framingham Heart Study.	n=2445 participants (6.2% prevalent AF and 6.6% incident AF), mean age 66.3 ± 8.9 years, 56.0% women. Median follow-up 5.4 years.	0.99-fold increased plasma concentrations in AF vs. SR (p=NR).<br>Plasma concentrations associated with incident AF.<br>Model 1: OR: 0.84, 95% CI: 0.73-0.97 (p=0.02).<br>Model 2: OR: 0.83, 95% CI: 0.72-0.97 (p=0.016).<br><br>Bonferroni $p=2.0\times10^{-4}$ (0.05/253 miRNAs).	Multivariable logistic regression. Model 1: age, sex, miR-processing.<br>Model 2: age, sex, miR-processing, height, weight, systolic blood pressure, diastolic blood pressure, current smoking, antihypertensive medication use, prevalent heart failure, myocardial infarction, and diabetes mellitus.
**miR-1274b**	Prospective population-based cohort study [20].	Participants who were enrolled in Framingham Heart Study.	n=2445 participants (6.2% prevalent AF and 6.6% incident AF), mean age 66.3 ± 8.9 years, 56.0% women. Median follow-up 5.4 years.	0.94-fold increased plasma concentrations in AF vs. SR (p=NR).<br>Plasma concentrations associated with incident AF.<br>Model 1: OR: 0.81, 95% CI: 0.69-0.96 (p=0.013).<br>Model 2: OR: 0.83, 95% CI: 0.71-0.99 (p=0.033).<br><br>Bonferroni $p=2.0\times10^{-4}$ (0.05/253 miRNAs).	Multivariable logistic regression. Model 1: age, sex, miR-processing.<br>Model 2: age, sex, miR-processing, height, weight, systolic blood pressure, diastolic blood pressure, current smoking, antihypertensive medication use, prevalent heart failure, myocardial infarction, and diabetes mellitus.

Abbreviations: AF, atrial fibrillation; CABG, coronary artery bypass graft; CHD, coronary heart disease; CI, confidence interval; HF, heart failure; HR, hazard ratio; n, number; NA, not available; NR, not reported; NS, not significant; OR, odds ratio; POAF, postoperative atrial fibrillation; SR, sinus rhythm.

Table S6. Association of previously-reported microRNAs with the odds of prevalent atrial fibrillation in our total study population and stratified by sex

Total Study Population			Men			Women		
**miRNA**	**OR (95% CI) ***	***p*-value**	**miRNA**	**OR (95% CI) ***	***p*-value**	**miRNA**	**OR (95% CI) ***	***p*-value**
let-7b-5p	0.98 (0.51-1.87)	0.941	let-7b-5p	0.82 (0.35-1.99)	0.658	let-7b-5p	1.06 (0.36-3.18)	0.918
let-7c-5p	0.85 (0.41-1.82)	0.672	let-7c-5p	0.95 (0.35-2.79)	0.921	let-7c-5p	0.58 (0.20-1.92)	0.359
let-7d-5p	0.90 (0.51-1.67)	0.741	let-7d-5p	0.69 (0.34-1.48)	0.317	let-7d-5p	1.27 (0.46-3.54)	0.652
RNU48-a2	NA	NA	RNU48-a2	NA	NA	RNU48-a2	NA	NA
miR-1	NA	NA	miR-1	NA	NA	miR-1	NA	NA
miR-10b-5p	0.71 (0.43-1.27)	0.222	miR-10b-5p	0.45 (0.23-0.93)	0.022	miR-10b-5p	1.14 (0.47-3.20)	0.797
miR-15	NA	NA	miR-15	NA	NA	miR-15	NA	NA
miR-15b-5p	1.05 (0.62-1.80)	0.848	miR-15b-5p	0.96 (0.50-1.93)	0.914	miR-15b-5p	1.22 (0.50-2.97)	0.657
miR-17-5p	1.04 (0.56-1.95)	0.903	miR-17-5p	0.82 (0.38-1.82)	0.623	miR-17-5p	1.49 (0.52-4.17)	0.455
miR-19a-3p	0.85 (0.51-1.38)	0.514	miR-19a-3p	0.86 (0.45-1.64)	0.643	miR-19a-3p	0.80 (0.34-1.75)	0.592
miR-20a-5p	1.30 (0.68-2.58)	0.436	miR-20a-5p	0.98 (0.45-2.27)	0.958	miR-20a-5p	2.20 (0.69-7.18)	0.187
miR-21	NA	NA	miR-21	NA	NA	miR-21	NA	NA
miR-21-5p	1.06 (0.69-1.63)	0.783	miR-21-5p	1.18 (0.66-2.12)	0.575	miR-21-5p	0.92 (0.46-1.80)	0.806
miR-23b-3p	0.82 (0.47-1.43)	0.486	miR-23b-3p	0.86 (0.42-1.81)	0.687	miR-23b-3p	0.75 (0.30-1.86)	0.540
miR-24-3p	1.04 (0.60-1.79)	0.881	miR-24-3p	1.22 (0.59-2.58)	0.591	miR-24-3p	0.81 (0.33-1.90)	0.643
miR-26a	NA	NA	miR-26a	NA	NA	miR-26a	NA	NA
miR-26a-5p	0.78 (0.48-1.27)	0.311	miR-26a-5p	0.70 (0.38-1.31)	0.248	miR-26a-5p	0.87 (0.36-2.04)	0.744
miR-27b-3p	0.84 (0.48-1.49)	0.534	miR-27b-3p	0.74 (0.37-1.58)	0.411	miR-27b-3p	0.97 (0.37-2.52)	0.944
miR-28-5p	0.91 (0.57-1.49)	0.711	miR-28-5p	1.14 (0.61-2.20)	0.690	miR-28-5p	0.78 (0.35-1.76)	0.550
miR-29a-3p	0.67 (0.35-1.29)	0.227	miR-29a-3p	0.57 (0.24-1.43)	0.220	miR-29a-3p	0.60 (0.20-1.75)	0.368
miR-29b	NA	NA	miR-29b	NA	NA	miR-29b	NA	NA
miR-29b-2-5p	NA	NA	miR-29b-2-5p	NA	NA	miR-29b-2-5p	NA	NA
miR-30a-5p	0.48 (0.23-1.02)	0.049	miR-30a-5p	0.34 (0.14-0.89)	0.023	miR-30a-5p	0.58 (0.17-2.25)	0.418
miR-30c-5p	0.70 (0.37-1.36)	0.272	miR-30c-5p	0.55 (0.26-1.25)	0.140	miR-30c-5p	0.97 (0.31-3.37)	0.964
miR-31-3p	NA	NA	miR-31-3p	NA	NA	miR-31-3p	NA	NA
miR-93-5p	0.85 (0.53-1.36)	0.505	miR-93-5p	0.80 (0.42-1.48)	0.476	miR-93-5p	0.99 (0.44-2.15)	0.976

miR-99b-5p	0.99 (0.55-1.82)	0.971	miR-99b-5p	0.62 (0.28-1.42)	0.252	miR-99b-5p	1.93 (0.75-5.39)	0.189
miR-100-5p	1.50 (0.94-2.47)	0.092	miR-100-5p	1.99 (1.09-4.00)	0.032	miR-100-5p	0.88 (0.41-2.04)	0.748
miR-106b-5p	1.09 (0.61-1.95)	0.776	miR-106b-5p	0.93 (0.45-1.97)	0.843	miR-106b-5p	1.35 (0.51-3.50)	0.543
miR-122-5p	1.42 (1.12-1.78)	0.003	miR-122-5p	1.77 (1.28-2.46)	0.001	miR-122-5p	1.07 (0.71-1.59)	0.731
miR-125a-5p	0.80 (0.45-1.50)	0.478	miR-125a-5p	0.41 (0.21-0.87)	0.015	miR-125a-5p	2.07 (0.71-6.39)	0.194
miR-125b-5p	1.55 (0.96-2.61)	0.080	miR-125b-5p	1.59 (0.87-3.13)	0.129	miR-125b-5p	1.60 (0.64-4.11)	0.323
miR-126	NA	NA	miR-126	NA	NA	miR-126	NA	NA
miR-126-3p	0.78 (0.49-1.27)	0.294	miR-126-3p	0.74 (0.40-1.44)	0.354	miR-126-3p	0.69 (0.32-1.59)	0.372
miR-126-5p	0.85 (0.59-1.26)	0.401	miR-126-5p	0.75 (0.47-1.23)	0.239	miR-126-5p	0.96 (0.50-1.96)	0.905
miR-133a	NA	NA	miR-133a	NA	NA	miR-133a	NA	NA
miR-134	NA	NA	miR-134	NA	NA	miR-134	NA	NA
miR-140-3p	NA	NA	miR-140-3p	NA	NA	miR-140-3p	NA	NA
miR-146a-5p	0.89 (0.68-1.15)	0.369	miR-146a-5p	0.88 (0.61-1.25)	0.470	miR-146a-5p	0.89 (0.59-1.33)	0.572
miR-148b-3p	0.95 (0.64-1.42)	0.791	miR-148b-3p	0.84 (0.49-1.46)	0.528	miR-148b-3p	1.09 (0.57-2.10)	0.797
miR-150-5p	1.02 (0.63-1.65)	0.948	miR-150-5p	0.88 (0.46-1.76)	0.706	miR-150-5p	1.16 (0.55-2.52)	0.699
miR-151a-3p	0.80 (0.49-1.32)	0.381	miR-151a-3p	0.65 (0.33-1.29)	0.214	miR-151a-3p	0.93 (0.43-2.01)	0.850
miR-152	NA	NA	miR-152	NA	NA	miR-152	NA	NA
miR-182-5p	NA	NA	miR-182-5p	NA	NA	miR-182-5p	NA	NA
miR-186-5p	0.60 (0.32-1.17)	0.114	miR-186-5p	0.58 (0.26-1.43)	0.202	miR-186-5p	0.49 (0.17-1.51)	0.197
miR-193a-5p	0.64 (0.39-1.03)	0.066	miR-193a-5p	0.49 (0.25-0.96)	0.041	miR-193a-5p	0.76 (0.35-1.68)	0.494
miR-196b-5p	NA	NA	miR-196b-5p	NA	NA	miR-196b-5p	NA	NA
miR-199a-3p	0.78 (0.50-1.20)	0.259	miR-199a-3p	0.66 (0.36-1.19)	0.169	miR-199a-3p	0.96 (0.49-1.88)	0.915
miR-200c-3p	NA	NA	miR-200c-3p	NA	NA	miR-200c-3p	NA	NA
miR-221-3p	0.86 (0.59-1.25)	0.446	miR-221-3p	0.82 (0.49-1.38)	0.468	miR-221-3p	0.90 (0.49-1.55)	0.705
miR-223-3p	0.84 (0.57-1.23)	0.371	miR-223-3p	0.77 (0.46-1.29)	0.314	miR-223-3p	0.99 (0.53-1.86)	0.971
miR-324-3p	0.88 (0.53-1.43)	0.602	miR-324-3p	1.08 (0.57-2.03)	0.810	miR-324-3p	0.61 (0.26-1.43)	0.263
miR-328	NA	NA	miR-328	NA	NA	miR-328	NA	NA
miR-331-3p	0.85 (0.53-1.40)	0.515	miR-331-3p	0.83 (0.45-1.59)	0.571	miR-331-3p	0.99 (0.44-2.30)	0.982
miR-339-5p	0.78 (0.47-1.29)	0.342	miR-339-5p	0.80 (0.39-1.61)	0.537	miR-339-5p	0.83 (0.37-1.78)	0.644
miR-342-3p	1.07 (0.58-2.01)	0.827	miR-342-3p	0.67 (0.30-1.58)	0.350	miR-342-3p	1.67 (0.63-4.32)	0.296
miR-363-3p	0.86 (0.53-1.45)	0.548	miR-363-3p	0.62 (0.35-1.19)	0.131	miR-363-3p	1.27 (0.53-3.46)	0.615
miR-375	0.88 (0.72-1.08)	0.206	miR-375	0.85 (0.66-1.10)	0.222	miR-375	0.92 (0.65-1.34)	0.658

miR-409-3p	0.92 (0.77-1.12)	0.401	miR-409-3p	0.92 (0.73-1.18)	0.498	miR-409-3p	0.91 (0.68-1.26)	0.568
miR-411-5p	NA	NA	miR-411-5p	NA	NA	miR-411-5p	NA	NA
miR-432	NA	NA	miR-432	NA	NA	miR-432	NA	NA
miR-483-5p	NA	NA	miR-483-5p	NA	NA	miR-483-5p	NA	NA
miR-484	0.90 (0.53-1.51)	0.697	miR-484	0.83 (0.41-1.63)	0.605	miR-484	0.99 (0.41-2.27)	0.988
miR-590	NA	NA	miR-590	NA	NA	miR-590	NA	NA
miR-720	NA	NA	miR-720	NA	NA	miR-720	NA	NA
miR-1274b	NA	NA	miR-1274b	NA	NA	miR-1274b	NA	NA

Abbreviations: CI, confidence interval; n, number; NA, not available; OR, odds ratio.

* Adjusted for age, cohort, body mass index, total cholesterol, high-density lipoprotein cholesterol, hypertension, smoking status, history of diabetes, history of coronary heart disease, history of heart failure, left ventricular hypertrophy on the electrocardiogram, use of cardiac medication, and use of lipid lowering medication (model 2).

Table S7. Association of previously-reported microRNAs with the risk of incident atrial fibrillation in our total study population and stratified by sex

Total Study Population			Men			Women		
**miRNA**	**HR (95% CI) ***	***p*-value**	**miRNA**	**HR (95% CI) ***	***p*-value**	**miRNA**	**HR (95% CI) ***	***p*-value**
let-7b-5p	0.82 (0.53-1.25)	0.353	let-7b-5p	0.97 (0.53-1.78)	0.916	let-7b-5p	0.63 (0.34-1.17)	0.145
let-7c-5p	0.89 (0.54-1.47)	0.641	let-7c-5p	0.97 (0.47-2.01)	0.943	let-7c-5p	0.74 (0.36-1.50)	0.396
let-7d-5p	0.93 (0.62-1.39)	0.704	let-7d-5p	0.93 (0.52-1.66)	0.812	let-7d-5p	0.90 (0.50-1.62)	0.736
RNU48-a2	NA	NA	RNU48-a2	NA	NA	RNU48-a2	NA	NA
miR-1	NA	NA	miR-1	NA	NA	miR-1	NA	NA
miR-10b-5p	0.85 (0.58-1.24)	0.406	miR-10b-5p	0.85 (0.48-1.51)	0.579	miR-10b-5p	0.81 (0.48-1.37)	0.432
miR-15	NA	NA	miR-15	NA	NA	miR-15	NA	NA
miR-15b-5p	1.02 (0.71-1.47)	0.903	miR-15b-5p	0.95 (0.57-1.57)	0.842	miR-15b-5p	1.10 (0.64-1.87)	0.738
miR-17-5p	0.76 (0.50-1.15)	0.193	miR-17-5p	0.71 (0.40-1.26)	0.239	miR-17-5p	0.79 (0.43-1.46)	0.454
miR-19a-3p	0.80 (0.57-1.12)	0.193	miR-19a-3p	0.67 (0.41-1.12)	0.125	miR-19a-3p	0.94 (0.59-1.50)	0.790
miR-20a-5p	0.94 (0.60-1.46)	0.772	miR-20a-5p	0.92 (0.49-1.71)	0.780	miR-20a-5p	0.88 (0.46-1.68)	0.691
miR-21	NA	NA	miR-21	NA	NA	miR-21	NA	NA
miR-21-5p	1.11 (0.83-1.48)	0.478	miR-21-5p	1.16 (0.76-1.76)	0.496	miR-21-5p	1.12 (0.76-1.66)	0.571
miR-23b-3p	0.88 (0.61-1.28)	0.512	miR-23b-3p	1.05 (0.62-1.79)	0.862	miR-23b-3p	0.73 (0.43-1.25)	0.252
miR-24-3p	0.85 (0.59-1.23)	0.390	miR-24-3p	0.94 (0.55-1.60)	0.813	miR-24-3p	0.76 (0.44-1.29)	0.302
miR-26a	NA	NA	miR-26a	NA	NA	miR-26a	NA	NA
miR-26a-5p	0.90 (0.64-1.27)	0.545	miR-26a-5p	0.99 (0.61-1.61)	0.961	miR-26a-5p	0.83 (0.50-1.36)	0.451
miR-27b-3p	0.79 (0.52-1.17)	0.238	miR-27b-3p	0.95 (0.52-1.78)	0.908	miR-27b-3p	0.62 (0.36-1.09)	0.096
miR-28-5p	1.05 (0.76-1.46)	0.763	miR-28-5p	1.25 (0.79-1.98)	0.343	miR-28-5p	0.90 (0.56-1.44)	0.656
miR-29a-3p	0.82 (0.52-1.29)	0.388	miR-29a-3p	0.98 (0.50-1.92)	0.955	miR-29a-3p	0.66 (0.34-1.26)	0.207
miR-29b	NA	NA	miR-29b	NA	NA	miR-29b	NA	NA
miR-29b-2-5p	NA	NA	miR-29b-2-5p	NA	NA	miR-29b-2-5p	NA	NA
miR-30a-5p	0.79 (0.45-1.40)	0.425	miR-30a-5p	1.13 (0.49-2.62)	0.771	miR-30a-5p	0.51 (0.23-1.12)	0.094
miR-30c-5p	0.84 (0.53-1.35)	0.477	miR-30c-5p	0.83 (0.44-1.58)	0.571	miR-30c-5p	0.83 (0.42-1.66)	0.599
miR-31-3p	NA	NA	miR-31-3p	NA	NA	miR-31-3p	NA	NA
miR-93-5p	0.71 (0.51-1.00)	0.047	miR-93-5p	0.65 (0.40-1.04)	0.073	miR-93-5p	0.74 (0.46-1.20)	0.223

miR-99b-5p	0.81 (0.55-1.19)	0.284	miR-99b-5p	0.87 (0.47-1.63)	0.670	miR-99b-5p	0.73 (0.44-1.21)	0.218
miR-100-5p	0.87 (0.63-1.22)	0.427	miR-100-5p	0.99 (0.59-1.63)	0.958	miR-100-5p	0.78 (0.50-1.20)	0.251
miR-106b-5p	0.83 (0.55-1.23)	0.346	miR-106b-5p	0.77 (0.45-1.34)	0.358	miR-106b-5p	0.83 (0.46-1.50)	0.532
miR-122-5p	0.91 (0.76-1.09)	0.304	miR-122-5p	0.75 (0.57-0.98)	0.036	miR-122-5p	1.07 (0.84-1.37)	0.587
miR-125a-5p	0.70 (0.47-1.03)	0.071	miR-125a-5p	0.81 (0.46-1.45)	0.487	miR-125a-5p	0.55 (0.31-0.97)	0.039
miR-125b-5p	0.91 (0.63-1.31)	0.599	miR-125b-5p	1.04 (0.59-1.82)	0.905	miR-125b-5p	0.76 (0.46-1.24)	0.274
miR-126	NA	NA	miR-126	NA	NA	miR-126	NA	NA
miR-126-3p	0.77 (0.55-1.08)	0.124	miR-126-3p	0.87 (0.0.53-1.43)	0.587	miR-126-3p	0.65 (0.40-1.05)	0.078
miR-126-5p	0.89 (0.68-1.16)	0.369	miR-126-5p	0.88 (0.61-1.27)	0.486	miR-126-5p	0.91 (0.61-1.34)	0.618
miR-133a	NA	NA	miR-133a	NA	NA	miR-133a	NA	NA
miR-134	NA	NA	miR-134	NA	NA	miR-134	NA	NA
miR-140-3p	NA	NA	miR-140-3p	NA	NA	miR-140-3p	NA	NA
miR-146a-5p	0.94 (0.79-1.12)	0.493	miR-146a-5p	0.96 (0.74-1.24)	0.740	miR-146a-5p	0.92 (0.73-1.17)	0.516
miR-148b-3p	0.93 (0.71-1.22)	0.607	miR-148b-3p	0.92 (0.62-1.38)	0.696	miR-148b-3p	0.92 (0.64-1.33)	0.653
miR-150-5p	0.98 (0.71-1.36)	0.922	miR-150-5p	1.13 (0.70-1.82)	0.631	miR-150-5p	0.87 (0.55-1.36)	0.534
miR-151a-3p	0.80 (0.57-1.11)	0.178	miR-151a-3p	0.95 (0.58-1.57)	0.847	miR-151a-3p	0.68 (0.44-1.06)	0.091
miR-152	NA	NA	miR-152	NA	NA	miR-152	NA	NA
miR-182-5p	NA	NA	miR-182-5p	NA	NA	miR-182-5p	NA	NA
miR-186-5p	0.95 (0.59-1.54)	0.839	miR-186-5p	1.09 (0.53-2.23)	0.822	miR-186-5p	0.85 (0.43-1.66)	0.628
miR-193a-5p	0.93 (0.67-1.28)	0.641	miR-193a-5p	0.70 (0.45-1.10)	0.120	miR-193a-5p	1.16 (0.73-1.86)	0.533
miR-196b-5p	NA	NA	miR-196b-5p	NA	NA	miR-196b-5p	NA	NA
miR-199a-3p	0.99 (0.73-1.33)	0.919	miR-199a-3p	1.04 (0.67-1.62)	0.852	miR-199a-3p	0.96 (0.63-1.45)	0.832
miR-200c-3p	NA	NA	miR-200c-3p	NA	NA	miR-200c-3p	NA	NA
miR-221-3p	0.93 (0.73-1.19)	0.569	miR-221-3p	1.04 (0.72-1.51)	0.833	miR-221-3p	0.84 (0.60-1.18)	0.309
miR-223-3p	0.95 (0.73-1.24)	0.704	miR-223-3p	1.01 (0.69-1.46)	0.979	miR-223-3p	0.92 (0.63-1.33)	0.645
miR-324-3p	0.70 (0.50-0.98)	0.038	miR-324-3p	0.73 (0.45-1.18)	0.197	miR-324-3p	0.60 (0.36-0.99)	0.044
miR-328	NA	NA	miR-328	NA	NA	miR-328	NA	NA
miR-331-3p	0.96 (0.69-1.33)	0.805	miR-331-3p	1.25 (0.78-2.02)	0.360	miR-331-3p	0.75 (0.47-1.18)	0.215
miR-339-5p	1.06 (0.77-1.47)	0.719	miR-339-5p	1.07 (0.67-1.70)	0.778	miR-339-5p	1.02 (0.65-1.59)	0.943
miR-342-3p	0.95 (0.63-1.44)	0.809	miR-342-3p	1.02 (0.53-1.96)	0.958	miR-342-3p	0.83 (0.48-1.45)	0.521
miR-363-3p	0.80 (0.56-1.13)	0.198	miR-363-3p	0.75 (0.46-1.22)	0.243	miR-363-3p	0.79 (0.48-1.30)	0.355
miR-375	0.94 (0.81-1.08)	0.376	miR-375	0.84 (0.70-1.01)	0.061	miR-375	1.07 (0.86-1.32)	0.549

miR-409-3p	0.93 (0.82-1.06)	0.263	miR-409-3p	0.92 (0.76-1.12)	0.392	miR-409-3p	0.91 (0.76-1.08)	0.260
miR-411-5p	NA	NA	miR-411-5p	NA	NA	miR-411-5p	NA	NA
miR-432	NA	NA	miR-432	NA	NA	miR-432	NA	NA
miR-483-5p	NA	NA	miR-483-5p	NA	NA	miR-483-5p	NA	NA
miR-484	0.78 (0.53-1.13)	0.192	miR-484	0.78 (0.45-1.35)	0.373	miR-484	0.73 (0.44-1.21)	0.218
miR-590	NA	NA	miR-590	NA	NA	miR-590	NA	NA
miR-720	NA	NA	miR-720	NA	NA	miR-720	NA	NA
miR-1274b	NA	NA	miR-1274b	NA	NA	miR-1274b	NA	NA

Abbreviations: CI, confidence interval; HR, hazard ratio; n, number; NA, not available.

* Adjusted for age, cohort, body mass index, total cholesterol, high-density lipoprotein cholesterol, hypertension, smoking status, history of diabetes, history of coronary heart disease, history of heart failure, left ventricular hypertrophy on the electrocardiogram, use of cardiac medication, and use of lipid lowering medication (model 2).
